# Cases from Practice

**Published:** 1889-12

**Authors:** Chas. L. Swift

**Affiliations:** Auburn, N. Y.


### CASES FROM PRACTICE.

(Read before the Cayuga County Homœopathic Medical Society.)

Was called at 10 P. M., to see a boy about seven years of age. He presented the following symptoms: Headache, backache, sore throat, chills and fever. The throat symptoms began on the left side, and were worse after sleeping. The throat was very sensitive to touch, and could bear nothing around it, as he said "he could not breathe." The tonsils looked purplish, tongue coated white, putrid breath; prostration.

The child seemed well at 8 P. M., when it went to bed.

Gave Lach.[30] in water; to be given every hour until there seemed to be improvement, then to stop the remedy.

Was sent for the next morning at 8 A. M., and found this condition of the patient: Tongue swollen, and the point of it protruding from the mouth. It was dry, and looked like a piece of burnt leather. There was a discharge from the nostrils, which seemed to burn and scald the face. The odor from the discharge and breath was very offensive. As often as he would drop off to sleep he would wake up and gasp for breath, and seem as if he was dying; could not examine the throat; all fluids seemed to escape through the nose.

My diagnosis was malignant diphtheria and recovery doubtful.

The symptoms seemed to point to *Lach.* as the remedy, and gave a dose of the M potency (B. & C.) dry on the tongue. Small lumps of ice was given it to keep the mouth moist.

Eleven A. M.—No worse.

Two P. M.—Not as much discharge from the nose, and the tongue less swollen.

Eight P. M.—Marked improvement; less discharge from the nose; can shut the mouth and drink a few sips of water; slept one hour with slight aggravation.

Nine P. M.—Much better; slept most of the time since midnight; no discharge from the nose; tongue looks more natural; can drink without difficulty; he continued to improve. Repeated the remedy once, when there seemed to be symptoms of paralysis.

Did the 30th produce any aggravations?

Mr. A., aged about fifty, was taken in the morning with cholera morbus, which grew so much worse at night that the family became frightened, and I was called at 11 P. M.

Found the patient bordering on a state of collapse; face pale and cold, eyes sunken and lustreless; the bowels moving frequently, rice-water evacuations, with griping cramps in the legs and cold sweat on the forehead. Gave Verat. and waited; no benefit.

Said he was dying, and bade the family good-bye; could not see; pulse very feeble; no control of the bowels; wants to be fanned.

Gave Carb. veg. dry on the tongue, and repeated it every fifteen minutes. Gave four doses before reaction set in. I remained until four in the morning. The next day gave a dose of China. He made a good recovery and a convert to Homœopathy.

Mr. F., aged thirty-two, red hair, blue eyes, painter by trade. Taken with spitting of blood after painting a cornice from a rope and pulley scaffold. The attack lasted one hour. He had frequent attacks for a week, and was under the care of a *regular* physician.

After an attack of unusual length and severity, I was called to the case. Patient had lost at least a quart of blood. He was pale, eyes glistening, and pulse quick. He was very restless, and was fearful, and knew he was going to die.

Gave Acon. Did not try to examine the chest, as he was bleeding, and I did not wish to irritate him any more.

Nine A. M.—Slept some during the night; had a slight attack toward morning. Soreness through the upper third of left lung and says he can feel the blood start from that part of the lung. Not so restless, and feeling better than the morning previous. Sac. lac.

Six P. M.—Severe coughing started another bleeding spell. Pressure across the chest; lies on the back or right side, coughs more on the left side. Phos.$^{30}$

No attack of bleeding for two days.

Third day another attack. Restless, constantly changing position; feels so tired all the time, although moving makes him feel better; dry cough, with tickling under the sternum; worse the fore part of the night; wants to be alone; tongue dry, red, with triangle on tip. Gave Rhus.$^{30}$

No more hemorrhages. Gained strength rapidly. Took a slight cold from getting damp, which was relieved by Phos.$^{30}$. In two weeks resumed his work.

CHAS. L. SWIFT, M. D.

AUBURN, N. Y.

